# Investigation of the Effects of Using Natural Fermented Lactic Acid Bacteria as Probiotics on Fattening Performance, Blood Parameters, and Intestinal Microflora in Broiler Chickens Under Heat Stress

**DOI:** 10.3390/vetsci13050488

**Published:** 2026-05-18

**Authors:** Sadık Serkan Aydin, Mehmet Avci, Nurcan Kirar, Ahmet Oruç, Mehmet Savrunlu, Aydin Daş

**Affiliations:** 1 Department of Animal Nutrition and Nutritional Disease, Faculty of Veterinary Medicine, Harran University, 63200 Şanlıurfa, Turkey; mavci@harran.edu.tr (M.A.); nurcankirar63@gmail.com (N.K.); 2 Department of Livestock, GAP Agricultural Research Institute, 63040 Şanlıurfa, Turkey; ahmet.oruc@tarimorman.gov.tr; 3 Şanliurfa Food Control Laboratory, Ministry of Food, Agriculture and Livestock, 63100 Şanlıurfa, Turkey; mehmet.savrunlu@tarimorman.gov.tr; 4 Department of Animal Husbandry, Faculty of Veterinary Medicine, Harran University, 63300 Şanlıurfa, Turkey; adas@harran.edu.tr

**Keywords:** heat stress, probiotic, cecum microbiology, broiler

## Abstract

Heat stress is a major environmental challenge in poultry production, negatively affecting growth performance, physiological functions, and intestinal health in broiler chickens. This study aimed to investigate the effects of probiotics prepared from meadow grass with molasses (PJFM) and commercial probiotics (TP) on fattening performance, blood parameters, and intestinal microflora in broiler chickens under heat stress. A total of 240 one-day-old Ross 308 broiler chicks (initial body weight: 46.72 ± 0.18 g) were used in a 42-day trial. The chicks were divided into six groups of 10 birds each, with four replicates. The study groups were divided into six groups: control (TNC) under normal environmental conditions (TN), PFJM (TNPJFM) and commercial probiotic (TNTP) supplemented in their drinking water, and control (HSC), HSPFJM, and HSTP under heat stress (HS) conditions. Temperature (34.5–36.2 °C) was applied for 42 days. Lactic acid bacteria (LAB) were 1.2 × 10^12^ cfu/mL in PFJM, and *Lactiplantibacillus plantarum* was the predominant species. Probiotics were added to the chicks’ drinking water at 0.5 mL/L. The control groups’ water was free of probiotics. This study revealed a decrease in body weight and body weight gain and a worse feed conversion ratio in the HSC group. The lowest carcass weight under heat stress was in the HSC group, and the highest was in the HSPFJM group. The lowest gizzard and highest bursa Fabricius percentages were in the probiotic-containing groups. Blood glucose and uric acid decreased in the HSPFJM group. In the HSC group, lactic acid bacteria (LAB) decreased, while *E. coli*, total mesophilic aerobic bacteria (TAB), enterobacteria, and yeast increased. In the probiotic-supplemented groups, LAB increased, while *E. coli*, yeast, and enterobacteria decreased. The study results suggest that PJFM supplementation may positively impact intestinal health in chickens exposed to heat stress, supporting growth performance and health parameters.

## Simple Summary

This study evaluates the effects of a naturally fermented probiotic prepared from meadow grass supplemented with molasses and a commercial probiotic on growth performance, blood parameters, and intestinal microflora in broiler chickens under normal and heat stress conditions. The poultry industry is a major contributor to global food production, and broiler chickens are highly valued for their rapid growth and efficient feed utilization. However, heat stress remains one of the most critical challenges, particularly in regions with high ambient temperatures or inadequate ventilation, leading to reduced productivity, impaired physiological functions, and disrupted intestinal health. Nutritional strategies such as probiotic supplementation have gained increasing attention as sustainable approaches to mitigate these negative effects. In particular, naturally derived probiotics obtained from plant-based fermentation processes offer a low-cost, environmentally friendly, and practical alternative to conventional commercial products. By evaluating these alternatives under both normal and stress conditions, this study contributes to the development of sustainable and effective strategies to improve poultry health and production efficiency.

## 1. Introduction

The poultry industry plays a pivotal role in global food production, with broiler chickens representing one of the most important sources of animal protein due to their rapid growth rate and high feed conversion efficiency. Despite these advantages, broilers are highly sensitive to environmental conditions. In particular, birds raised in hot climates or under inadequate ventilation are frequently exposed to heat stress, which represents a major challenge for sustainable poultry production [1]. Stressful environmental conditions activate various physiological defense mechanisms through hormonal and neural pathways. The first responses to stress usually occur in the neuroendocrine and immune systems. In this process, the hypothalamic–pituitary–adrenal (HPA) axis and the sympathoadrenal system are activated, leading to a series of physiological reactions that help the body maintain homeostasis. However, the strength and duration of these responses vary depending on the intensity and duration of the stressor. Among the various environmental stressors encountered in poultry production, heat stress is considered one of the most detrimental factors affecting broiler performance. Temperatures exceeding the optimal thermal comfort range for broilers (18–22 °C) lead to increased body temperature and elevated respiratory rate as birds attempt to dissipate excess heat [2,3]. Prolonged exposure to high ambient temperatures results in a range of physiological and metabolic disturbances, including reduced feed intake, impaired growth performance, weakened immune function, and disruption of intestinal barrier integrity [4,5]. Thermal stress disrupts intestinal integrity and leads to significant alterations in the cecal microbiota. These changes are characterized by an increased abundance of the phylum Firmicutes and the genus Tyzzerella, along with a reduction in the phylum Bacteroidetes and the genera Bacteroides, Parabacteroides, and Romboutsia [4]. Similarly, Zhang et al. [6] reported that thermal stress significantly modified gut microbial composition, resulting in decreased populations of beneficial bacteria such as Lactobacillus spp. and Bifidobacterium spp., while increasing the abundance of potentially pathogenic bacteria including Salmonella spp., Escherichia coli, and Clostridium spp. In addition to microbial alterations, thermal stress also impairs intestinal morphology. Structural changes include epithelial cell shedding, disruption of the villus–crypt architecture, reduced villus height, inflammatory cell infiltration, and edema in the lamina propria [4]. Furthermore, thermal stress affects intestinal metabolism. Total short-chain fatty acids (SCFAs) and several metabolites—including propionate, butyrate, fumarate, malate, lactate, succinate, β-alanine, niacin, aspartate, and ethanolamine—were reported to decrease, whereas concentrations of fructose and azelaic acid were markedly increased [7]. These alterations ultimately compromise both animal welfare and production efficiency. To mitigate the negative effects of heat stress, various nutritional and management strategies have been explored. In recent years, increasing attention has been directed toward the use of probiotics, particularly those containing lactic acid bacteria (LAB), as a promising approach to improve poultry health and productivity under stressful conditions [8,9]. Previous studies have demonstrated the effectiveness of naturally fermented lactic acid bacteria (LAB) in poultry. For example, Aydın, S. S. and Hatipoğlu, D. [8], as well as Hatipoğlu, D. et al. [9], administered fermented LAB preparations to one-day-old broiler chicks at a dose of 0.5 mL/L via drinking water for a period of 42 days. As a result, improvements were reported in body weight gain, feed conversion ratio, gut microbiota composition, and hormonal balance. These findings indicate that the use of fermented LAB provides significant biological and practical advantages in poultry. Furthermore, the doses used in these studies (0.5 mL/L) are consistent with those applied in the present study, supporting the suitability of the selected dosage. Lactobacillus species, in particular, are known for their ability to inhibit pathogenic microorganisms through competitive exclusion, production of antimicrobial compounds, and modulation of the intestinal environment. Moreover, multi-strain probiotic formulations may enhance gut microbial diversity and stability, thereby increasing the resilience of the intestinal microbiota against environmental stress and pathogenic infections [10,11]. Probiotic microorganisms provide beneficial effects through several mechanisms. They can attach to intestinal epithelial cells, grow rapidly in the gastrointestinal tract, and prevent the colonization of harmful bacteria through competitive inhibition. By occupying space on the intestinal surface, probiotics reduce the ability of pathogens to attach and multiply. At the same time, they help stimulate the host’s immune response and maintain the balance of the intestinal microbiota [12]. In addition to commercial probiotic products, naturally fermented microorganisms obtained from plant sources have gained increasing interest as potential probiotic alternatives. Probiotic bacteria adhere to intestinal epithelial cells, proliferate rapidly, and are not absorbed from the digestive tract. Thus, probiotic bacteria colonize the gut surface, preventing the adhesion and proliferation of pathogens that are not natural members of the intestines, thereby enhancing the immune response [13]. Commercial probiotic products are often costly, and their effectiveness may decline during extended storage due to reductions in viable cell counts. These limitations have encouraged the search for alternative probiotic sources. Fermented lactic acid bacteria (LAB) liquids represent a promising alternative, as they are of natural biological origin and commonly contain a diverse community of both homofermentative and heterofermentative LAB species.

Naturally fermented LAB preparations have gained increasing attention as potential alternatives to conventional commercial probiotics. Such products are easy and inexpensive to produce and apply, safe, non-toxic, and environmentally friendly. In addition, multi-strain compositions can promote synergistic interactions among microbial species, enhancing gut microbiota stability and functional benefits. In the present study, PFJM was selected as a natural probiotic source due to its rich and diverse microbial composition, predominantly consisting of LAB, and its potential to provide broader functional benefits compared to single-strain commercial probiotics. Unlike conventional probiotic products, PFJM can be produced easily under field conditions using locally available materials, making it cost-effective and sustainable. Its multi-strain structure may enhance gut health, immune response, and performance, particularly under stress conditions such as heat stress. These characteristics make PFJM a promising candidate for practical applications in poultry production systems. When plant-derived microorganisms are fermented in the presence of carbon-rich substrates such as molasses under suitable environmental conditions, microbial growth is enhanced and diverse bacterial communities can develop. This fermentation process facilitates the isolation and enrichment of beneficial bacterial strains with probiotic potential. Lactic acid bacteria isolated from molasses-based fermentation systems possess several beneficial characteristics. These include strong acid-producing capacity, tolerance to environmental stress conditions, and the ability to produce short-chain fatty acids that support intestinal health. Due to these functional properties, such microorganisms have considerable potential to be used as effective probiotic candidates [14]. Therefore, the present study aimed to evaluate the effects of a probiotic derived from molasses-supplemented meadow grass fermentation (PJFM) on growth performance, blood biochemical parameters, and intestinal microbiota composition in broiler chickens raised under both normal and heat stress conditions.

## 2. Materials and Methods

All chicks used in the study were individually weighed and placed in pens at a stocking density of 10 chicks per square meter. The chicks were randomly allocated into six experimental groups. Each group consisted of four replicates with 10 chicks per replicate. Thus, each experimental group included a total of 40 chicks. The chicks used in the study were a mixture of female and male chicks. This sample size is commonly accepted in broiler performance research and considered sufficient for reliable statistical analysis. In total, 240 one-day-old Ross 308 broiler chicks were used in the experiment. Throughout the experimental period, the chicks were fed starter, grower, and finisher diets appropriate to their age. The diets were formulated based on corn, soybean meal, and wheat according to NRC [15] recommendations.

In this study, heat stress was not confirmed by direct physiological measurements but was assessed based on behavioral indicators such as panting, wing spreading, and reduced activity. These signs, together with the observed decline in growth performance, indicate that heat stress was successfully induced.

The composition and nutritional contents of the feed mixtures used in the study are presented in detail in Table 1. The experimental group design and the treatments applied are given in Table 2.

### 2.1. Experimental Groups

The following table provides details of the experimental groups.

### 2.2. Preparation of Fermented Lactic Acid Bacteria (LAB) Liquid

The fermented LAB liquid used in the study was prepared with modifications based on the method of Masuko et al. [16] to increase bacterial density. The meadow grass used for preparing the fermented liquid in this study was harvested from the fields within the campus of Harran University Faculty of Veterinary Medicine during the mid-vegetation stage. The pH value of the fresh plant material was 6.44, while the lactic acid bacteria count was determined to be 7 × 10^4^ cfu/mL. For this purpose, 1000 g of fresh plant material (meadow grass) was mixed with 1000 mL of sterile water and blended for 2 min. The resulting plant liquid mixture was filtered through double-layered cheesecloth and transferred to sterile falcon tubes; then, 5% molasses was added, and the mixture was incubated at 30 °C for 5 days. The fermented LAB cultures obtained after incubation were stored at 0–4 °C during the trial period. The pH value of the fermented liquid obtained after incubation was 3.76, and the lactic acid bacteria count was 1.2 × 10^12^ cfu/mL. Probiotics (TP and PFJM) were applied to the chicks’ drinking water at a concentration of 0.5 mL/L. This dosage was chosen based on previous studies [8,9], which reported beneficial effects on growth performance and gut health at similar concentrations. The applied dose ensures a sufficiently high number of viable lactic acid bacteria (LAB) per milliliter, supporting effective intestinal colonization and competitive exclusion of pathogenic bacteria. Biologically, this concentration maximizes probiotic activity without causing adverse effects. Economically, it allows for cost-effective application in broiler production, balancing efficacy and affordability.

Probiotic solutions (PFJM and TP) were removed from refrigeration immediately prior to administration to ensure microbial viability. For water administration, the solutions were thoroughly mixed to achieve homogeneity, and the daily intake per bird was estimated based on average water consumption. Furthermore, the stability of the probiotics in water was monitored by evaluating lactic acid bacteria counts throughout the administration period.

**Figure 1:**
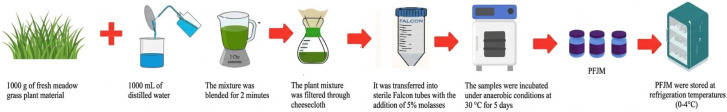
Flowchart for the preparation of fermented lactic acid bacteria (LAB) liquid.

**Figure 2:**
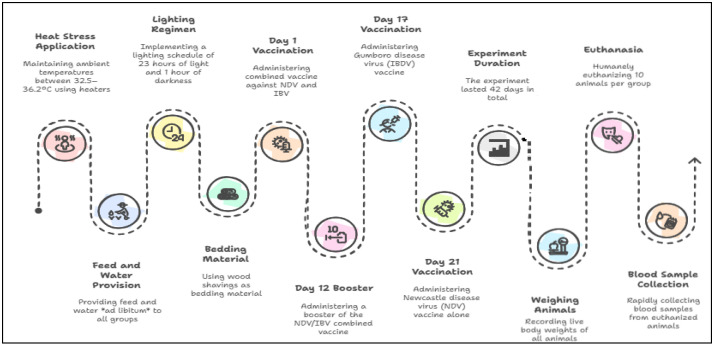
Experimental application flowchart.

The workflow diagram illustrating the preparation process of the fermented lactic acid bacteria (LAB) liquid is shown in Figure 1. The process diagram presenting the overall flow of the experimental application protocol is provided in Figure 2.

### 2.3. Experimental Applications

In this study, heat stress was induced using heaters to maintain the ambient temperature in the stress group compartments between 34.5 and 36.2 °C throughout the experimental period. In contrast, birds in the thermoneutral control group were reared under standard temperature management conditions recommended for broiler production. Accordingly, the ambient temperature was gradually reduced with age and maintained at 33 °C during the first week, 30 °C in the second week, 27 °C in the third week, 24 °C in the fourth week, 21 °C in the fifth week, and 18 °C in the sixth week. During the trial period, all groups were freely provided with feed and water, and a lighting program was implemented with 23 h of light and 1 h of darkness. Wood shavings were used as bedding material. The total duration of the trial was 42 days. On day 1, the chicks were vaccinated with a combined vaccine containing Newcastle disease and Infectious Bronchitis viruses. The same vaccine was administered again on the 12th day of the trial. On the 17th day, a vaccine containing Gumboro viruses was applied, and a vaccine containing only Newcastle viruses was administered on the 21st day.

### 2.4. Data Collection, Slaughter, and Determination of Carcass Organ Weights

The live weight gain, feed consumption, and feed conversion ratios of the groups were measured weekly. On the 42nd day of the trial, all animals were individually weighed, and their live weights were recorded. Following the weighing, blood samples were quickly taken from 10 randomly selected broilers from each group after slaughter. The birds were processed using an automatic plucking machine to remove feathers, their feet were removed, and the internal organs were extracted from the intestines. The wings, back, neck, thighs, and breast meat of each bird were weighed separately, and the obtained values were evaluated in relation to the pre-slaughter live weight. The internal organs obtained from the animals slaughtered on the 42nd day, including the liver, heart, spleen, bursa Fabricius, and gizzard, were weighed, and the weight of each organ was recorded individually. The gizzard was weighed after being cleaned of surrounding tissue and fat and emptied. Organ weights were also analyzed proportionally based on live weight. In addition, the carcass yield was calculated by determining the ratio of the hot carcass weight obtained after the removal of internal organs to the pre-slaughter live weight. These data were used to assess carcass quality.

### 2.5. Microbiological Analyses

The microbiota analysis of the fermented natural lactic acid bacteria (PFJM) was performed using the targeted shotgun sequencing method with the latest MinKNOW version of the Mk1C device to exclusively analyze bacteria. This was conducted using the Oxford Nanopore Technologies NBD114.96 amplicon protocol. Previously established workflows were followed for 16S rRNA-targeted metagenomic analysis. Amplicon libraries were generated using a primer pair targeting an approximately 1400 bp fragment encompassing the V1–V9 regions of the bacterial 16S rRNA gene. To enable sequencing on the Oxford Nanopore Technologies (ONT) platform, Nanopore barcode adapter sequences were incorporated at the 5′ ends of the target-specific primers. The 16S rRNA-specific primer–adapter sequences were as follows: forward primer: 5′-TTTCTGTTGGTGCTGATATTGC-AGRGTTTGATYHTGGCTCAG-3′; reverse primer: 5′-ACTTGCCTGTCGCTCTATCTTC-TACCTTGTTAYGACTT-3′. PCR amplification was performed using a Proofreading DNA Polymerase 2× Reaction Mix with 200 nM of each primer. The amplification reactions were carried out in a thermal cycler under the following conditions: an initial denaturation at 95 °C for 3 min, followed by 25 cycles of denaturation at 95 °C for 30 s, annealing at 55 °C for 30 s, and extension at 72 °C for 90 s, with a final extension at 72 °C for 5 min. The resulting PCR products were verified by electrophoresis on agarose gel to confirm the expected amplicon size (~1450 bp). Subsequently, the amplified products were purified using a PCR Product Purification Kit according to the manufacturer’s instructions prior to downstream library preparation and sequencing.

To determine the total lactic acid bacterial count, 1 mL was taken from each of the previously conducted serial dilutions and inoculated into sterile Petri dishes. Subsequently, De Man, Rogosa, and Sharpe Agar (MRSA) (Merck) was added to these plates, and microbiological inoculation was carried out using the pour plate method. Following this, the plates were incubated under anaerobic conditions at 37 °C for 24–48 h. After incubation, all developed colonies were counted [17]. For the enumeration of coliform bacteria, the pour plate method was used. In this method, 1 mL from suitable dilutions was inoculated into sterile Petri plates, and 15 mL of Violet Red Bile (VRB, Merck) agar, cooled to 45–50 °C, was added to ensure thorough homogenization. After solidification, an additional 5 mL of sterile VRB agar was poured. After fully solidifying, it was incubated at 37 °C for 1 day. At the end of the incubation, colonies that were dark red with a diameter of 0.5–2 mm and typically formed reddish halos around them were counted as typical coliforms [18,19]. For the enumeration of *Enterobacteriaceae* bacteria, the prepared dilutions were inoculated onto Violet Red Bile Glucose Agar (Merck 1.10275, Darmstadt, Germany) and incubated at 30 ± 1 °C for 48 h. At the end of the incubation, colonies that were 1–2 mm in diameter, were red, and formed halo shapes around them underwent the oxidase test (Merck 1.13300, Darmstadt, Germany). Typical colonies that yielded negative results were counted [20]. For yeast and mold counting, ISO 21527-1:2008 [21] instructions were followed. For this study, 0.1 mL samples were taken from all dilutions prepared as previously described and spread onto Dichloran Rose Bengal Chloramphenicol (DRBC) Agar. Then, the plates were incubated under aerobic conditions at 25 °C for 5 days, and colony counting was performed after incubation [22]. The *E. coli* count was performed using the ISO 16649-2 [23]. method. Serial dilutions were prepared, and inoculations were made on TBX (Tryptone Bile X-Glucuronide, Oxoid CM 0945, Basingstoke, UK) agar, followed by incubation at 37 °C for 24 h. Metallic green colonies that formed were counted and evaluated [22]. The total mesophilic aerobic count was performed by inoculating the prepared dilutions onto sterile Petri dishes using the pour plate method with Plate Count Agar (PCA) (Merck, 1.05463, Darmstadt, Germany) and incubating at 30 ± 1 °C for 3 days. Petri plates with visible colonies were assessed. The colonies growing on PCA were counted and multiplied by the dilution factor, and the total aerobic mesophilic bacteria (TAB) count was calculated [24].

### 2.6. Biochemical Analyses

Biochemical parameters such as total protein, albumin, glucose, cholesterol, triglycerides, HDL cholesterol, LDL cholesterol, VLDL cholesterol, uric acid, and alkaline phosphatase (ALP) were examined in serum samples obtained from a total of 60 broiler chicks slaughtered on the 42nd day of the trial. Analyses were quantitatively performed using Roche brand commercial kits on a Roche Integra 800 autoanalyzer by spectrophotometric methods at Şanlıurfa Güven Laboratories.

### 2.7. Statistical Analysis

The experiment was conducted as a 2 × 3 factorial arrangement in a completely randomized design, with environmental condition (thermoneutral vs. heat stress) and drinking water supplementation (control, commercial probiotic, and PFJM) as fixed factors. Data were analyzed using two-way ANOVA (SPSS, version [25]), including both main effects and interaction effects. Assumptions of normality and homogeneity of variance were verified using the Shapiro–Wilk and Levene’s tests, respectively, and all variables met the requirements for parametric analysis (*p* > 0.05). When significant interaction effects were observed, the effect of probiotic supplementation was evaluated within each environmental condition separately to interpret the interactions. Post hoc mean comparisons were performed using Duncan’s multiple range test when significant differences were detected. Statistical significance was declared at *p* < 0.05.

## 3. Results

**Figure 3:**
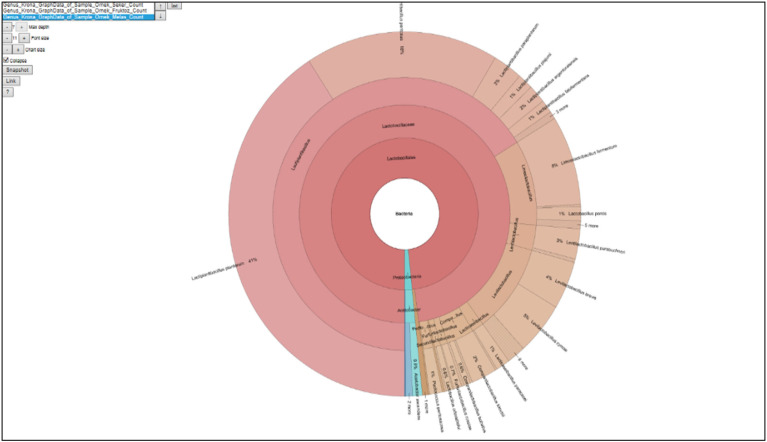
Graph of the taxonomic microbial composition of the PJFM sample.

In this study, data regarding the microbial and chemical properties of PJFM obtained from the fermentation of grass forage are presented in Table 3. The weekly live weights of the broiler chicks used in the research are shown in Table 4, live weight gains in Table 5, feed consumptions in Table 6, and feed conversion ratios in Table 7. Findings related to carcass characteristics and internal organ weight percentages are presented in Table 8, while data on serum biochemical parameters are summarized in Table 9. The levels of microbial and chemical parameters of the intestinal microflora are summarized in Table 10. The correlation between organic acid concentrations and gut microbiota composition is shown in Table 11. Additionally, the distribution plot of the taxonomic microbial composition of the PJFM sample is presented in Figure 3, visually illustrating the structure of the microbial community. In the tables (Table 4, Table 5, Table 6, Table 7, Table 8, Table 9 and Table 10), the values highlighted in yellow represent the comparison of the overall statistical mean values between normal environmental conditions (TN) and heat stress conditions (HS). The values highlighted in green indicate the statistical mean values of the control (C), commercial probiotic (TP), and PFJM groups across both environmental conditions (TN and HS). The values highlighted in blue represent the statistical mean values of the control (C), commercial probiotic (TP), and PFJM groups under normal environmental conditions (TN). The values highlighted in red indicate the mean values of the control (C), commercial probiotic (TP), and PFJM groups under heat stress conditions (HS).

When examining Table 4 for average live weight (LW) values of the groups, it was observed that a homogeneous distribution among the experimental groups was achieved by considering the live weights of the chicks at the beginning of the study (*p* > 0.05). Significant differences were found between the groups in the live weight measurements during the 1st and 2nd weeks of the trial (*p* < 0.05). During these weeks, the highest live weight values were obtained in the groups supplemented with PJFM under both normal and temperature stress conditions. No significant difference was found between the groups in terms of live weight values during the weighings conducted at the beginning of the study and in the 3rd, 4th, 5th and 6th weeks (*p* > 0.05). However, looking at the live weight averages obtained at the end of the 6th week, the average live weight was determined to be 2478.30 g in groups under normal environmental conditions, while it was 2395.97 g in groups subjected to temperature stress. In the 6th week of the study, the highest live weight values in both environmental conditions were measured in the groups supplemented with PJFM; under normal conditions, the value was 2516.28 g, and under temperature stress, it was 2469.56 g. Under temperature stress, the control group’s live weight was measured at 2307.53 g, which was the lowest value, while the commercial probiotic (HSTP) group weighed 2418.77 g, and the HSPFJM group weighed 2469.56 g. These findings indicate that probiotic supplementation administered to chicks under heat stress had a positive effect on live weight, especially in the PJFM group.

When examining Table 5 for average live weight gain (LWG) values of the groups, it was observed that both temperature stress and probiotic addition had positive effects on live weight gain (LWG) during the 1st and 2nd weeks of the study. However, in the 3rd, 4th, 5th, and 6th weeks, there was no significant effect of probiotic applications on live weight gain under normal conditions, while positive effects were identified in groups subjected to temperature stress. Evaluating the 1–6-week period, it was determined that live weight gain decreased under temperature stress, while the addition of probiotics increased live weight gain. During this period, the lowest live weight gain under temperature stress was observed in the control group (HSC) at 2260.52 g, while the highest value was recorded at 2424.38 g in the HSPFJM-supplemented group.

When examining the feed consumption values in Table 6, significant differences in feed consumption among the groups were found during the 2nd, 3rd, and 4th weeks of the study (*p* < 0.05). In the HSC and HSPFJM-supplemented groups, feed consumption showed a marked increase during the 2nd and 4th weeks due to temperature stress; however, a decrease was recorded in the 6th week. In contrast, the HSTP-supplemented group showed an increase in feed consumption throughout all weeks. These findings indicate that the TP probiotic exhibited a stronger effect in maintaining feed intake and increasing overall consumption compared to the other groups, despite heat stress. In the 1st week of the study, chicks exposed to temperature stress showed improved feed conversion compared to those under normal environmental conditions. However, during the 3rd and 4th weeks, temperature stress created adverse effects on feed conversion, with significant differences found in the feed conversion rate (FCR) values among the experimental groups. Particularly in the 5th week (5 HF), the use of HSPFJM under temperature stress had a significant effect on FCR values. During the same period, the feed conversion rate in the control group (HSC) was recorded at 2.58, while it was 2.33 in the HSTP group and 2.05 in the HSPFJM group. Furthermore, it was observed that temperature stress had a significant effect on feed conversion over the 1–6-week evaluation period (1–6 HF), while the effects of probiotics during this time were not significant. During this period, the best feed conversion rate was achieved in the HSPJM group at 2.00, while it was 1.95 in the TNPFJM group.

When examining Table 8 regarding the average pre-slaughter live weights, carcass weights, and internal organ weight percentages of the groups, no differences were detected among the groups in terms of carcass weight percentages, except for slaughter weight and breast weight percentage (*p* > 0.05). Differences between the groups regarding slaughter weight and breast weight percentage were found to be statistically significant (*p* < 0.05). In terms of slaughter live weight, it was observed that temperature stress and probiotic use had a significant effect. Under both normal and temperature stress environmental conditions, the lowest carcass weight was identified in the control group, while the highest value was found in the PFJM-supplemented group.

When examining the internal organ weight percentages in broiler diets under normal and temperature environmental conditions, no statistically significant differences were found among groups regarding liver weight percentage (*p* > 0.05). However, differences among groups regarding gizzard, heart, spleen, and bursa Fabricius weight percentages were statistically significant (*p* < 0.05). Upon examining gizzard percentages, it was observed that probiotic applications had a distinct effect under temperature stress conditions. While the gizzard percentage increased in the control group under temperature stress, it decreased in the probiotic-supplemented groups. The highest gizzard percentage was recorded at 1.56% in the control group, while the lowest percentage was determined to be 1.14% in the HSPFJM-supplemented group. As a result of temperature stress, the heart percentage decreased, with the lowest heart percentage recorded at 0.56% in the HSPFJM-supplemented group. The spleen percentage decreased in both the control (HSC) and HSTP-supplemented groups due to temperature stress, whereas it maintained its value in the PFJM-supplemented group. In our study, the bursa Fabricius percentage in the control group decreased due to temperature stress. Evaluations regarding the bursa Fabricius percentages also indicated that probiotics were effective against temperature stress. The lowest bursa Fabricius percentage was found to be 0.09% in the control (HSC) group, while the highest value was recorded at 0.14% in the HSPFJM-supplemented group. Compared to normal environmental conditions, a decreasing trend in the bursa Fabricius percentage was observed under temperature stress in the control and TP-supplemented groups, excluding the PFJM-supplemented group. In our study, the lowest carcass weight was noted in the control group, while the highest value was observed in the PFJM group. This situation demonstrates the supportive effect of probiotics on growth performance.

When examining the blood biochemical parameter values of the groups in the study, as shown in Table 9, it was found that the total cholesterol, HDL, LDL cholesterol, and VLDL values of the control and experimental groups were not affected by the addition of probiotics. However, statistically significant differences were observed among the glucose, ALP, and uric acid values in the groups receiving probiotics. When comparing normal environmental conditions to temperature stress conditions, a decrease in blood glucose levels was noted. The lowest glucose level in the blood was identified in the HSTP-supplemented group, with a value of 218.29 under temperature stress conditions. Regarding ALP values, under normal environmental conditions, the highest value of 2713 was observed in the TNTP group, while the lowest value of 1869.00 was found in the TNPFJM-supplemented group. Under temperature stress, the lowest ALP values were identified in the HSTP- and HSPFJM-supplemented groups, with values of 1962.86 and 1975.75, respectively. Sugiharto et al. [21] reported that the addition of probiotics containing Bacillus strains at concentrations of 0.1%, 0.5%, and 1% to the broiler diet had no significant effect on total cholesterol, HDL cholesterol, and LDL cholesterol values. These results are consistent with the findings of our study.

Heat stress (HS) and probiotic (P) treatments have shown significant differences between groups regarding duodenum and cecum pH, cecum total aerobic bacteria (TAB), lactic acid bacteria (LAB), Enterobacter, coliforms, *Escherichia coli* (*E. coli*), yeast, and mold, as well as levels of lactic acid (LA), acetic acid (AA), and propionic acid (PA) in the cecum (*p* < 0.05).

When examining the pH, microbial populations, and organic acid levels of the gut microbiota of the groups in the study, as presented in Table 10, it was found that duodenum pH values ranged from 6.42 to 6.65, while cecum pH values ranged from 7.28 to 7.65. In the TP group under normal environmental conditions, the pH value increased under temperature stress conditions, whereas in the PJFM-supplemented group, the pH value decreased during temperature stress.

Total mesophilic aerobic bacteria (TAB) count increased due to the addition of probiotics under normal environmental conditions. However, in groups exposed to temperature stress, the TAB count decreased with the probiotic addition. The lowest TAB value was determined in the HSPFJM-supplemented group at Log_10_ 9.04 cfu/mL. Species-level identification was performed exclusively for the fermented lactic acid bacteria (LAB) liquid. In contrast, microbiological analyses of cecal samples were conducted at the group and family levels rather than at the species level. These analyses included the enumeration of total lactic acid bacteria, total mesophilic aerobic bacteria, Enterobacteriaceae, coliforms, Escherichia coli, yeasts, and molds.

The count of lactic acid bacteria (LAB) decreased under temperature stress compared to normal environmental conditions. The LAB count increased with the addition of probiotics under both normal and temperature stress conditions. Under normal environmental conditions, the highest LAB count was observed in the TNTP-supplemented group at Log_10_ 10.83 cfu/mL, while under temperature stress, the highest value was measured in the HSPFJM-supplemented group at Log_10_ 10.81 cfu/mL. In the control and TP-supplemented groups, a decrease in LAB count was noted due to temperature stress, while an increase was observed in the PFJM-supplemented group. Generally, there was a trend of increased Enterobacter counts under temperature stress. The lowest Enterobacter count was determined in the TP-supplemented group under both environmental conditions. In terms of coliform bacteria counts, the lowest value under normal environmental conditions was recorded in the PFJM-supplemented group at Log_10_ 5.34 cfu/mL. *E. coli* counts decreased due to probiotic treatments under both normal and temperature stress conditions. In groups exposed to temperature stress, the highest *E. coli* count was measured in the control (HSC) group at Log_10_ 6.64 cfu/mL, while the lowest values were found in the HSTP group at Log_10_ 3.90 cfu/mL and in the HSPFJM group at Log_10_ 3.99 cfu/mL. Yeast counts were also affected by environmental conditions, showing a decrease with the addition of probiotics under both normal and temperature stress conditions. The highest yeast counts were observed in the control group under both environmental conditions, while the lowest value among the temperature-stressed groups was determined in the HSPFJM-supplemented group at Log_10_ 2.04 cfu/mL.

The amount of lactic acid (LA) in the cecum content decreased due to temperature stress in the control and TP-supplemented groups, while it increased in the PFJM-supplemented group. The highest LA level was measured in the TNTP-supplemented group under normal environmental conditions at 62.45. The level of acetic acid (AA) decreased across all groups due to temperature stress. The highest AA amount was determined in the TNTP-supplemented group under normal environmental conditions at 33.55. The level of propionic acid (PA) reached its highest value in the TP-supplemented group among both normal and temperature-stressed groups.

## 4. Discussion

In this study, the number of lactic acid bacteria (LAB) in the fermented natural LAB liquids prepared by adding 5% molasses and incubating for 5 days was found to be 1.2 × 10^12^ cfu/mL. Aydın and Denek [26] reported LAB counts in fermented liquids prepared from alfalfa with added sucrose at levels of 1–5%, ranging from 3.3 × 10^8^ to 1.15 × 10^9^ cfu/mL. In a study conducted by Aydın and Denek [27], a LAB count of 2.53 × 10^12^ cfu/mL was obtained after 13 days of incubation with 10% sucrose addition, which is similar to our findings. Xie et al. [14] noted that the addition of molasses to mixtures prepared from various plants significantly increased the LAB count. The LAB count of 1.2 × 10^12^ cfu/mL obtained in our study supports the notion that the use of molasses as a nutrient source, along with appropriate incubation times and temperatures, could achieve this value. Prior studies have noted that the LAB count associated with pre-plant species can vary from 1 × 10^1^ to 1.0 × 10^7^ cfu/mL, highlighting differences in the number and types of LAB present. Factors contributing to these differences include plant species, ultraviolet radiation, ambient temperature, environmental humidity, and other aspects related to the plants, as the breakdown of the plants increases the bacterial count they carry [28,29]. This is supported by the LAB count of 7 × 10^4^ cfu/mL in our fresh grass forage.

Mutuş et al. [30] and Yeter and Altun [31] reported that probiotic supplementation did not significantly affect growth performance parameters such as body weight in broiler chickens, which is consistent with the data we obtained under normal environmental conditions. Under such conditions, where there are no threats of disease or stress, probiotic additions may not have a positive effect on growth performance. If an optimal microbiota balance already exists in the intestines, the addition of probiotics may not make a significant difference since the advantages provided by probiotics are already present [32]. The effect of probiotics can vary based on factors including the type and number of bacteria in the probiotic product, the rate of addition to the feed, the breed of the animal, slaughter age, feed content, and many environmental factors [32]. Our findings of improvements in body weight on days 7 and 14 (*p* < 0.05) align with the results of Serin H. [33]. This situation may originate from the positive effects of probiotics on the microbiota during the initial development of the gastrointestinal flora [33].

The literature suggests that high-temperature stress negatively affects broilers’ growth. Studies by Liu et al. [4] and Aydın and Hatipoğlu [8] reported a significant decrease in weight gain under heat stress, which is consistent with the results of our study. Heat stress can negatively affect the composition and stability of the intestinal microbiota. However, organic acids and other antimicrobial metabolites produced by lactic acid bacteria (LAB) may help mitigate these adverse effects. By lowering the intestinal pH, these metabolites create an unfavorable environment for pathogenic microorganisms and enhance competitive exclusion against them. As a result, the proliferation of pathogenic bacteria is suppressed, contributing to the stabilization of the microbial community and a reduction in pathogen load. Numerous studies have shown that probiotic applications mitigate the adverse effects of heat stress. Both natural and commercial probiotics have been reported to enhance growth performance and reduce stress-related losses in broilers exposed to heat stress [8,9]. The reduction in the negative effects of heat stress by the PFJM probiotic in our study could be attributed to the microbial species present in the fermented liquids. Probiotics contribute to the maintenance of microbial homeostasis by modulating the balance between beneficial and pathogenic microorganisms in the gut microbiota, favoring the proliferation of beneficial bacteria. This modulation improves intestinal microbial stability and enhances nutrient utilization. Consequently, improved feed efficiency leads to better growth performance in broiler chickens. The PFJM fermented liquid exhibited a rich microbial profile predominantly comprising Lactiplantibacillus species, especially 41% from *L. plantarum*. Additionally, various other lactic acid bacteria (LAB) genera such as Limosilactobacillus, Levilactobacillus, and Pediococcus were also found. The high proportion of *Lactiplantibacillus plantarum* strains in PFJM is believed to significantly increase body weight gain in broilers by positively modulating gut morphology and immune response [34,35,36,37]. Furthermore, the presence of different LAB strains in PFJM may create a synergistic effect by producing short-chain fatty acids and other metabolites that support growth. Despite high-temperature stress, the PFJM liquid obtained in our study exhibited similar or greater effects on body weight gain compared to commercial probiotics. This finding indicates that the use of naturally sourced probiotic mixtures can improve broiler performance under heat stress. Probiotic bacteria colonize by implanting in intestinal epithelial cells and are not absorbed from the digestive tract. Thus, they inhibit the implantation and proliferation of disease-causing pathogenic bacteria that are not natural residents of the intestines by adhering to intestinal epithelial cells [38]. As a result, probiotics enhance weight gain by preventing diseases, allowing the gut flora to return to normal and promoting healthy development in animals. The increase in LAB counts among beneficial bacteria and the reduction in *E. coli*, a harmful pathogen, in the intestines due to probiotic supplementation [38] support the increase in live weight.

Feed consumption (FC) and the feed conversion ratio (FCR) are fundamental performance indicators that reflect the growth efficiency of broiler chickens. Probiotics are known to stimulate the secretion of digestive enzymes like phytase, amylase, and protease, which are crucial for food digestion and absorption [39]. This enzymatic activity is important for increasing feed utilization in poultry. The various lactic acid bacterial species in PFJM, particularly *Lactiplantibacillus plantarum* at 41%, along with *Limosilactobacillus fermentum* and *Levilactobacillus zymae*, may stabilize the intestinal mucosa, enhance enzyme activities, and optimize digestion and absorption. This synergistic effect is likely more pronounced than the improvement provided by the TP treatment. Differences in feed conversion efficiency observed in poultry studies may be attributed to variations in environmental temperature and the nutrient composition of the diets used. The previous literature has consistently shown that probiotic supplementation improves weight gain and the feed conversion ratio in poultry [40,41]. For instance, Liao et al. [41] reported that the application of *Lactobacillus plantarum* improved feed utilization and reduced appetite loss due to stress, while Song et al. [42] showed that a probiotic mixture significantly decreased FCR in heat-stressed chickens. Tunç, MA.’s [43] study on heat-stressed broilers determined the feed conversion ratio to be in the range of (1.78–1.96), while Tuğalay, ÇŞ et al. [44] reported it to be in the range of (1.89–1.96). The feed conversion ratios are similar to those of our study subjects. In our study, the group with the best FCR after the 1–6-week feeding period was the PFJM-supplemented group. Probiotics can improve broiler growth performance and health by enhancing gut morphology and increasing the ratio of beneficial bacteria to pathogenic bacteria [45]. The better feed conversion ratio observed in the PFJM-supplemented group can also be explained by an increase in LAB counts among beneficial bacteria in the cecum, while also reducing *E. coli* counts among pathogenic bacteria [46].

As reported by Jamshidparvar et al. [47] and Sjofjan et al. [48], probiotic supplementation increases both body weight and carcass yield in broilers. Soumeh et al. [49] also reported that probiotics significantly increased the relative weights of the liver, spleen, gizzard, bursa, and carcass yield, while some studies have reported limited or insignificant effects on carcass weight. Many studies have demonstrated that heat stress reduces carcass weight and live weight [48,49,50]. The bursa of Fabricius weight was maintained in the PFJM-supplemented group. This condition serves as a key indicator of the immunoprotective capacity of immune organs. Some higher values in the PFJM group suggest that probiotics somewhat mitigate the negative effects of stress.

In our data on gizzard percentage, the highest value was found in the control (HSC) group at 1.56%, while the lowest was in the HSPFJM group at 1.14%. While Sjofjan et al. [48] reported that probiotic applications increased gizzard weight, other studies have reported no significant changes in the weights of the gizzard and similar organs. The lower gizzard percentage in the PFJM group, despite a higher live weight ratio, is likely due to a reduction in relative organ weight based on the increase in body weight. Acid-tolerant bacteria such as Lactobacillus dominate gizzard microbiota, contributing to grinding feed, although microbial activity is limited compared to other segments. The bacterial species in the gizzard include Lactobacilli, Enterococci, and Enterobacteria [51]. The high proportion of Lactobacillus in the PFJM structure can be explained by the improvement in gastrointestinal health and the lower gizzard percentage due to better nutrient absorption [51].

Chronic heat stress leads to significant reductions in the indices of the bursa Fabricius and spleen [52]. In this context, the higher bursa Fabricius ratio obtained in the PFJM group can be attributed to the protective effects of probiotic applications on immune organs, which is consistent with findings reported by Soumeh et al. [49]. Their study demonstrated that probiotic supplementation increased the relative weight of the bursa Fabricius, similar to our findings. The weight of immune organs is considered an important indicator of the health status of the immune system in poultry [53]. Probiotics have been reported to increase the lymphocyte counts in lymphoid organs, leading to an increase in the weight of these organs [54,55]. Therefore, the increase in the weights of the spleen and bursa Fabricius in our study is considered a significant and valuable finding, indicating that probiotics strengthen the immune system in chickens exposed to heat stress. The preservation of the bursa Fabricius in probiotic-treated groups despite temperature stress suggests that probiotics may mitigate the immunosuppressive effects of stress. The bursa Fabricius is a primary lymphoid organ critical for the development of B lymphocytes (B cells) in poultry. B cells play an important role in humoral immunity through antibody production. B cells that mature in the bursa Fabricius enter circulation and settle in secondary lymphoid organs like the spleen, where they encounter antigens and are activated to produce antibodies. Stress can impair immune function by causing body weight loss and reducing the functional activity of the bursa of Fabricius [7]. Heat stress, in particular, induces oxidative damage in immune organs and leads to a reduction in their relative organ indices. Previous studies have shown that heat stress can severely damage the morphology of the thymic cortex and the bursa of Fabricius, thereby affecting the proliferation and functional development of T and B lymphocytes and ultimately leading to immune dysfunction in broiler chickens. Prolonged or severe heat stress can also disrupt the regulation of the immune system by the neuroendocrine system. Under such conditions, the immune response may become dysregulated and excessively activated, resulting in systemic inflammation. This inflammatory response may reduce the relative weights of immune-related organs, including the thymus, bursa, spleen, and liver, consequently impairing overall immune function [56]. Furthermore, heat stress can compromise intestinal barrier integrity. When the intestinal barrier is disrupted, gut bacteria and their metabolites, such as endotoxins, may translocate into the bloodstream. This process can further stimulate systemic inflammatory responses and exacerbate multiple organ dysfunction [57]. Probiotics may help counteract these adverse effects by stabilizing the intestinal microbiota. They suppress harmful microorganisms, promote the growth of beneficial bacteria, and stimulate the secretion of immunomodulatory compounds. Through these mechanisms, probiotics may influence the neuroendocrine system via the microbiota–gut–brain axis. While the percentages of the spleen and bursa Fabricius decrease in the control (HSC) group due to heat stress, increases in the PFJM probiotic group indicate that the modulation of gut microbiota may indirectly influence the functions of the spleen and bursa Fabricius by activating immune cells and cytokine production [58]. The percentage of the bursa Fabricius was highest in the PFJM group (0.14%) and lowest in the control (HSC) group (0.09%). It is known that heat stress leads to shrinkage in lymphoid organs such as the thymus, spleen, and bursa. The findings by Hashemitabar and Hosseinian [59] regarding lower spleen weights (−36% and −50%) and bursa Fabricius weights (−30% and −43%) due to heat stress align with our results. Probiotics added to drinking water significantly increased the weight of immune organs compared to the HS group. Based on these findings, the report that Bacillus and Lactobacillus probiotics reduce the negative effects of heat stress on the health of immune organs supports our study [54].

In our study, significant differences were observed in blood glucose, uric acid, and ALP (alkaline phosphatase) levels in groups receiving probiotics. While the literature frequently indicates that heat stress increases blood glucose [5], it has also been reported that glucose levels may decrease under prolonged or severe stress conditions [60]. In our study, blood glucose levels in groups under temperature stress were found to be low, with the lowest glucose level measuring 218.29 mg/dL in the HSTP-supplemented group. This finding parallels the results of Zulkifli et al. [61], who reported that chronic heat stress reduced blood glucose levels. Furthermore, the literature indicates that probiotics exhibit a balancing effect on increased glucose levels under stress conditions. Indeed, Zulkifli et al. [61] demonstrated that probiotic-supplemented drinking water reduced elevated glucose levels under high temperatures and decreased subsequent corticosteroid release. In our study, the higher percentage of the bursa Fabricius under temperature stress due to probiotic addition compared to the control group is significant for immunity.

Heat-induced disruption of intestinal microbiota balance can elevate the release of endotoxins, particularly lipopolysaccharide (LPS), by pathogenic bacteria, increasing intestinal permeability and facilitating bacterial translocation and endotoxemia. This endotoxemia may cause long-term liver injury and, in some cases, reduced liver mass [62]. The principal mechanisms of probiotics include improving the composition of the intestinal microbiota, strengthening the integrity of the intestinal barrier, and reducing LPS translocation to suppress systemic endotoxemia [63]. Moreover, probiotics have been reported to enhance the production of short-chain fatty acids (SCFAs), contributing to the regulation of immune responses [64,65]. Through these mechanisms, inflammatory activation is reduced, and antioxidant defense systems are strengthened. Regarding heat-stress-associated liver injury, probiotics help by mitigating oxidative stress, inflammation, and fibrogenesis; their use can induce apoptosis and cell-cycle arrest in hepatic stellate cells and Kupffer cells, thereby reducing liver injury and fibrosis. Stress hormones (such as cortisol) have the potential to increase glucose production. If the bursa Fabricius is healthy, immune responses can occur more effectively and be balanced, which may assist in regulating glucose levels. Immune cells utilize glucose as an energy source. When immune system activity increases, the demand for glucose by immune cells also rises. The healthy function of the bursa Fabricius supports the development of these cells and positively impacts overall metabolism, which may indirectly help balance blood glucose levels and support our findings. Regarding ALP values, the lowest value under normal environmental conditions was found in the TNPFJM group, while the lowest ALP levels under heat stress were recorded in the HSPFJM and HSTP groups. Since alkaline phosphatase is generally an indicator of liver health, it is suggested that probiotic application alleviates liver stress. The liver plays a pivotal role in regulating metabolic processes in broiler chickens. Under stress conditions caused by high temperatures, clear increases in ALP levels can occur. The highest liver percentages found in probiotic-supplemented groups during heat stress in our study indicate that probiotics increase digestive efficiency by regulating gut microbiota, thereby improving nutrient absorption and reducing the burden on the liver, leading to decreased ALP levels. Significant differences were found in uric acid and triglyceride levels in the probiotic-treated groups. Uric acid is not only the final product of nitrogen excretion in poultry but also considered an indicator of protein metabolism and antioxidant capacity. Some studies have reported that certain probiotic treatments may counteract this effect and increase uric acid levels. For example, Hasan et al. [66] reported that probiotic application increased uric acid levels in chickens under heat stress. In our study, the significant increase in uric acid levels due to probiotic supplementation may be explained by a potential antioxidant response mechanism or involvement in protein digestion. Oxidative damage and inflammation caused by heat stress can lead to increased uric acid production. Impairments in the metabolic functions of the liver and the kidneys’ inadequate excretion of uric acid result in elevated serum uric acid levels. Probiotics reduce unnecessary uric acid production by enhancing the efficiency of protein digestion. More protein corresponds to less ammonia and therefore lower uric acid levels. Probiotics enhance digestive efficiency by increasing the number of beneficial bacteria in the intestines. They aid in the more effective breakdown and absorption of proteins, which can subsequently reduce uric acid production. Probiotics maintain gut health by minimizing the impact of harmful bacteria. A healthier gut ensures more effective absorption of proteins, which consequently may decrease uric acid production. Regarding triglyceride levels, there are conflicting findings in the literature. Some studies have reported that heat stress increases triglyceride levels [5], which is consistent with our results. Conversely, some studies have reported that heat stress decreases triglyceride levels [67,68].

Based on the study findings, it is understood that the observed decrease in pH in the cecum under heat stress, despite PFJM supplementation, is not due to LA, PA, and AA [59]. In contrast, the rise in intestinal pH in the control group due to temperature stress aligns with our results. The literature indicates that this increase in intestinal pH in chickens exposed to heat stress may arise from imbalances in gut microbiota and increases in pathogenic microorganisms following heat stress [69]. In a study conducted by Siriken et al. [70], a reduction in coliform counts with probiotic application and an increase in LAB and Enterobacter counts were observed, which resemble the microbial changes obtained in our study following PJFM application. Additionally, in a study by Siriken et al. [70] conducted under normal environmental conditions, increases in total aerobic bacteria (TAB), LAB, and Enterobacter counts were observed due to probiotic application, while no significant changes in coliform counts were detected. These findings align with the microbial changes we obtained following PJFM application. In a study by Mookiah et al. [71], it was found that the addition of lactic acid bacteria to broiler diets increased LAB populations in the cecum and suppressed *E. coli*, which is similar to data obtained in our study under normal environmental conditions. The reduction in *E. coli*, a pathogen, in both environmental conditions can be explained by the capacity of probiotics to adhere to the intestinal epithelium, occupying binding sites for pathogens, competing for nutrients, and producing compounds such as lactic acid, hydrogen peroxide, and bacteriocins that inhibit pathogen growth [72]. In this context, it was observed that probiotic applications in the PFJM and TP groups led to a reduction in *E. coli* and other enteric pathogens while preserving and increasing LAB counts [72,73,74], consistent with our study’s findings. In our research, the relatively high pathogen load in the non-probiotic control group under heat stress was expected. However, it is important to note that we observed an increase in LAB counts in the PFJM probiotic groups despite heat stress. This finding indicates that the addition of probiotics mitigates negative effects such as dysbiosis, pathogen proliferation, and barrier disruption triggered by temperature stress. Natural probiotics have shown a more pronounced increase in LAB counts in broilers exposed to heat stress, suggesting that naturally sourced probiotics may generally be more resilient to temperature stress. The highest LAB count under heat stress was observed in the HSPFJM group, supporting the statements by Aydın and Hatipoğlu [8].

Cecal microbial populations are indicators of gut health in animals [75]. The cecum represents a complex microbial colonization ecosystem in poultry. Volatile fatty acids are generally produced via bacterial fermentation in the cecum, which is essential for gut function and integrity [76]. The lowest levels of LA, AA, and PA under both environmental conditions were found in association with the addition of the PFJM probiotic. We assess that this decrease is due to probiotics altering the proportion of bacteria in the cecal microflora that produce AA, PA, and LA. In a study by Novak et al. [77], it was shown that some volatile fatty acids decreased in the cecum of meat chickens receiving probiotics, supporting our results. Strong correlations between Enterobacteriaceae counts and concentrations of acetic, propionic, and butyric acids were observed in the cecum of chickens by van der Wielen et al. [32]. The higher counts of enterobacter in the control group compared to the PFJM-supplemented group under both environmental conditions and the associated high AA and PA levels are aligned with the findings of van der Wielen et al. [32]. The low volatile fatty acids in the PFJM-supplemented group suggest a change in the cecal microbiota. In complex ecosystems like the cecum, LAB can dominate numerically over other microorganisms. However, deliberate downregulation of metabolic activity has been observed as a survival strategy for LAB in high-competition environments [78,79]. In environments with high competition, enzymes and metabolic pathways that LAB attempt to produce may be suppressed by other microorganisms, reducing the fermentation capacity of LAB. Such interactions affect not only LAB metabolism but also the overall microbial balance in the ecosystem. While LAB attempt to survive against inhibitory factors through their defense mechanisms, their metabolic rate may also be reduced, leading to diminished fermentative performance. In highly competitive environments, other microorganisms may produce antibiotic-like substances or enzyme inhibitors that limit LAB’s growth and fermentative capabilities [80]. In our study, despite the high lactic acid count in the PFJM-supplemented group, the presence of low fatty acids in the cecum can be explained by this report.

The beneficial effects of probiotic supplementation observed in this study can be explained through interconnected physiological, microbial, and immunological mechanisms. From a microbial perspective, the PFJM probiotic enriched the gut microbiota with beneficial lactic acid bacteria, particularly *Lactiplantibacillus plantarum*, while suppressing pathogenic bacteria such as *E. coli*. This shift in microbial balance enhances competitive exclusion, stabilizes intestinal ecology, and improves barrier integrity. At the physiological level, improved microbial balance promotes digestive enzyme activity, nutrient absorption, and gut morphology, ultimately enhancing feed efficiency and growth performance. Additionally, the production of antimicrobial metabolites and short-chain fatty acids contributes to maintaining intestinal pH and limiting pathogen proliferation. From an immunological standpoint, probiotics support the development and function of immune organs such as the bursa Fabricius and spleen by stimulating lymphocyte activity and modulating cytokine responses. This immune modulation is particularly critical under heat stress conditions, where oxidative stress and inflammation impair immune function.

Furthermore, these mechanisms are interconnected through the microbiota–gut–brain axis. By stabilizing gut microbiota, probiotics may reduce stress-induced endocrine responses, improve metabolic regulation (e.g., glucose and uric acid balance), and alleviate systemic inflammation. Collectively, these integrated pathways explain how probiotic supplementation mitigates the negative effects of heat stress and enhances overall broiler performance.

## 5. Conclusions

PJFM, a naturally fermented probiotic derived from meadow grass, improves growth performance in broiler chickens under heat stress by enhancing live weight, weight gain, and the feed conversion ratio. Probiotic supplementation modulates blood biochemical parameters, including glucose, ALP, and uric acid levels, suggesting supportive effects on metabolism and liver function. Additionally, PJFM positively influences gut microflora, increasing beneficial lactic acid bacteria while reducing pathogenic bacteria, thereby contributing to intestinal health and microbial stability. The probiotic supports immune organ health, including the bursa Fabricius and spleen, indicating potential immunoprotective effects under stress conditions. Its low cost, safety, and environmental friendliness highlight its practical value as an alternative to commercial probiotics in broiler production. Future studies should explore optimal PJFM dosages, application durations, combinations with prebiotic components, and effects under varying environmental stresses such as humidity or airflow to further optimize performance, gut health, and overall well-being in poultry.

## Figures and Tables

**Table 1:** Composition of feed mixtures and nutritional contents used.

Nutritional Components (%)	Starter	Grower	Finisher
Vegetable Oil	2.90	4.20	5.30
Wheat, Winter	2.00	2.78	1.50
Corn, Yellow	52.65	53.78	57.00
Fish Meal, 72% HP	2.87	1.83	-
Soybean Meal, 48%	35.65	33.65	33.00
Dicalcium Phosphate	1.90	1.70	1.70
DL-Methionin	0.24	0.20	0.22
Limestone	0.84	0.82	0.77
L-Lysine	0.23	0.23	0.23
L-Threonine	0.11	0.11	0.07
Sodium Bicarbonate	0.20	0.20	0.20
Salt	0.20	0.20	0.20
Vitamin–Mineral Mixture	0.30	0.30	0.30
Analyzed and Calculated Values (%)			
Dry Matter (DM, %):	89.90	89.80	90.30
Crude Protein (CP, %):	23.00	21.50	20.00
Metabolizable Energy (ME, kcal/kg)	3000	3101	3200

**Table 2:** Arrangement of experimental groups and treatments applied.

Group No.	Treatment	Number of Animals Used in the Trial	Heat Stress	Drinking Water Supplement
1	Control (Thermoneutral)/TNC	40	No	No
2	Commercial Probiotic (Thermoneutral)/TNTP	40	No	0.5 mL/L Commercial Probiotic
3	PFJM (Thermoneutral)/TNPFJM	40	No	0.5 mL/L PFJM
4	Control (Heat Stress)/HSC	40	Yes—Heat Stress (34.5–36.2 °C)	No
5	Commercial Probiotic (Heat Stress)/HSTP	40	Yes—Heat Stress (34.5–36.2 °C)	0.5 mL/L Commercial Probiotic
6	PFJM (Heat Stress)/HSPFJM	40	Yes—Heat Stress (34.5–36.2 °C)	0.5 mL/L PFJM

TNC: control group under normal environmental conditions; TNTP: commercial probiotics under normal environmental conditions; TNPFJM: probiotics obtained by adding 5% molasses to grass forage and incubating for 5 days under normal environmental conditions; HSC: control group under temperature environmental conditions; HSTP: commercial probiotics under temperature environmental conditions; HSPFJM: probiotics obtained by adding 5% molasses to grass forage and incubating for 5 days under temperature environmental conditions; PFJM: probiotic derived from grass forage using molasses as a substrate to obtain fermented natural lactic acid bacteria.

**Table 3:** Total LAB count (LAB), yeast, mold, pH, lactic acid (LA), and acetic acid (AA) values of PJFM and the microbial composition of commercial probiotics.

Parameter	Fresh Plant Liquid Mixture	Fermented Liquid After 5 Days of Incubation
Total LAB Count	7 × 10^4^ cfu/mL	1.2 × 10^12^ cfu/mL
Yeast	2.9 × 10^3^ cfu/mL	5.5 × 10^7^ cfu/mL
Mold	1.8 × 10^4^ cfu/mL	<10 cfu/mL
pH	6.44	3.76
Lactic Acid	20 g/kg DM	685 g/kg DM
Acetic Acid	24 g/kg DM	102 g/kg DM
After adding 5% molasses to the plant liquid mixture obtained from the grass forage and incubating for 5 days, the microbial composition of the fermented liquid (PFJM) is as follows: Lactiplantibacillus: 66.215% (with *Lactiplantibacillus plantarum* constituting 41%). Limosilactobacillus: 10.112% (with *Limosilactobacillus fermentum* constituting 7%). Levilactobacillus: 10.628% (with *Levilactobacillus zymae* constituting 5%). Lentilactobacillus: 2.982% (with *Lentilactobacillus parabuchneri* constituting 3%). Companilactobacillus: 3.362% (with *Companilactobacillus kimchii* constituting 2%). Lacticaseibacillus: 1.445%. Furfurilactobacillus: 0.865%. Secundilactobacillus: 0.76%. Acetobacter: 1.503%. Pediococcus: 1.173%. Weissella: 0.526%. Lapidilactobacillus: 0.134%. Enterobacter: 0.103%. Microbial species and amounts in commercial probiotics (TP): *Lactobacillus plantarum*: 1.2 × 10^8^ cfu/mL. *Lactobacillus acidophilus*: 3.8 × 10^8^ cfu/mL. *Lactobacillus salivarius*: 6.4 × 10^8^ cfu/mL. *Bacillus subtilis*: 1.8 × 10^8^ cfu/mL.		

**Table 4:** Average live weight (LW) values of the groups.

	Beginning	1 HF	2 HF	3 HF	4 HF	5 HF	6 HF
TN	46.97	164.64	399.90	789.90	1279.38	1825.90	2478.30
HS	46.50	172.16	427.39	792.63	1268.42	1780.95	2395.97
C	47.03	169.12	396.31	780.52	1263.80	1777.69	2406.29
TP	47.11	162.43	414.22	783.65	1269.30	1791.37	2420.33
PFJM	46.05	173.49	430.16	810.92	1290.57	1847.33	2494.14
TNC	47.06	167.48	367.12	776.12	1275.81	1833.21	2498.16
TNTP	47.03	154.89	407.10	781.00	1275.52	1796.71	2421.79
TNPFJM	46.83	171.83	427.60	814.05	1287.28	1848.68	2516.28
HSC	47.00	170.87	427.70	785.25	1250.88	1718.00	2307.53 ^b^
HSTP	47.21	170.56	421.90	786.51	1262.59	1785.62	2418.77 ^ab^
HSPFJM	45.18	175.33	433.00	807.44	1294.22	1845.83	2469.56 ^a^
SEM	0.17	1.18	3.25	6.08	9.56	14.81	19.13
*p*-value							
HS	0.1370	0.0020	0.0000	0.8260	0.5900	0.1480	0.0370
P	0.0550	0.0010	0.0000	0.0920	0.4750	0.1230	0.1330
HS*P	0.0640	0.0500	0.0010	0.8610	0.7920	0.2220	0.1080

a, b: Values with different letters in the same column were found to be different; HF: week; TN: averages under normal environmental conditions; HS: averages under temperature stress conditions; C: control group averages; TP: averages of commercial probiotics; PFJM: averages of probiotics obtained by adding 5% molasses to grass forage and incubating for 5 days; TNC: control group under normal environmental conditions; TNTP: commercial probiotics under normal environmental conditions; TNPFJM: probiotics obtained by adding 5% molasses to grass forage and incubating for 5 days under normal environmental conditions; HSC: control group under temperature environmental conditions; HSTP: commercial probiotics under temperature environmental conditions; HSPFJM: probiotics obtained by adding 5% molasses to grass forage and incubating for 5 days under temperature environmental conditions. *: interaction; P: effect of probiotics (TP and PFJM); HS*P: interaction between heat stress conditions and probiotics.

**Table 5:** Average live weight gain (LWG) values of the groups.

	1 HF	2 HF	3 HF	4 HF	5 HF	6 HF	1–6 HF
TN	117.67	235.26	389.99	489.49	546.51	652.40	2431.32
HS	125.66	255.23	365.23	475.79	512.53	615.03	2349.47
C	122.08	227.20	384.20	483.28	513.89	628.60	2359.26
TP	115.32	251.79	369.43	485.64	522.07	628.96	2373.22
PFJM	127.44	256.67	380.76	479.64	556.76	646.82	2448.10
TNC	120.43	199.63	409.00	499.70	557.40	664.95	2451.11
TNTP	107.86	252.21	373.90	494.52	521.19	625.07	2374.76
TNPFJM	125.00	255.77	386.45	473.23	561.40	667.60	2469.45
HSC	123.87	256.83	357.55	465.63	467.13	589.53	2260.52 ^b^
HSTP	123.35	251.34	364.62	476.08	523.03	633.15	2371.56 ^ab^
HSPFJM	130.15	257.67	374.44	486.78	551.61	623.72	2424.38 ^a^
SEM	1.05	2.30	3.38	4.25	6.11	5.84	18.99
*p*-value							
HS	0.0000	0.0000	0.0000	0.1270	0.0080	0.0020	0.0370
P	0.0000	0.0000	0.1990	0.8800	0.0100	0.3770	0.1230
HS*P	0.0390	0.0000	0.0170	0.0700	0.0040	0.0130	0.1030

a, b: Values with different letters in the same column were found to be different; HF: week; TN: averages under normal environmental conditions; HS: averages under temperature stress conditions; C: control group averages; TP: averages of commercial probiotics; PFJM: averages of probiotics obtained by adding 5% molasses to grass forage and incubating for 5 days; TNC: control group under normal environmental conditions; TNTP: commercial probiotics under normal environmental conditions; TNPFJM: probiotics obtained by adding 5% molasses to grass forage and incubating for 5 days under normal environmental conditions; HSC: control group under temperature environmental conditions; HSTP: commercial probiotics under temperature environmental conditions; HSPFJM: probiotics obtained by adding 5% molasses to grass forage and incubating for 5 days under temperature environmental conditions; *: interaction; P: effect of probiotics (TP and PFJM); HS*P: interaction between heat stress conditions and probiotics.

**Table 6:** Average feed consumption (FC) values of the groups.

	1 HF	2 HF	3 HF	4 HF	5 HF	6 HF	1–6 HF
TN	178.09	401.24	794.11	1059.12	1219.51	1223.78	4875.86
HS	159.58	441.81	845.11	1143.02	1199.93	1205.91	4995.33
C	177.68	419.26	799.81	1092.56	1214.49	1203.53	4907.33
TP	171.75	418.59	831.98	1100.66	1217.93	1238.21	4979.11
PFJM	157.08	426.73	827.04	1109.98	1196.74	1202.79	4920.34
TNC	185.20	400.95	786.75	1060.73	1223.53	1232.28	4889.50
TNTP	171.75	385.05	793.40	1050.10	1215.83	1231.88	4848.00
TNPFJM	177.33	417.73	802.18	1066.53	1219.18	1207.18	4890.08
HSC	170.15	437.58	812.88	1124.40	1205.45	1174.78	4925.15
HSTP	171.75	452.13	870.55	1151.23	1220.03	1244.55	5110.23
HSPFJM	136.83	435.73	851.90	1153.43	1174.30	1198.40	4950.60
SEM	7.54	3.42	8.49	5.98	9.30	7.17	29.32
*p*-value							
HS	0.2350	0.0000	0.0080	0.0000	0.3070	0.2290	0.0570
P	0.5290	0.5690	0.2750	0.5060	0.6160	0.0970	0.5770
HS*P	0.5520	0.0280	0.4850	0.4510	0.5690	0.1520	0.2510

HF: week; TN: averages under normal environmental conditions; HS: averages under temperature stress conditions; C: control group averages; TP: averages of commercial probiotics; PFJM: averages of probiotics obtained by adding 5% molasses to grass forage and incubating for 5 days; TNC: control group under normal environmental conditions; TNTP: commercial probiotics under normal environmental conditions; TNPFJM: probiotics obtained by adding 5% molasses to grass forage and incubating for 5 days under normal environmental conditions; HSC: control group under temperature environmental conditions; HSTP: commercial probiotics under temperature environmental conditions; HSPFJM: Probiotics Obtained By Adding 5% Molasses To Grass Forage And Incubating For 5 Days Under Temperature Environmental Conditions; *: interaction; P: effect of probiotics (TP and PFJM); HS*P: interaction between heat stress conditions and probiotics.

**Table 7:** Average feed conversion rate (FCR) values of the groups.

	1 HF	2 HF	3 HF	4 HF	5 HF	6 HF	1–6 HF
TN	1.57	1.85	1.99	2.14	2.21	1.86	1.97
HS	1.29	1.71	2.33	2.40	2.32	1.94	2.10
C	1.49	2.03	2.10	2.26	2.39	1.90	2.08
TP	1.54	1.66	2.24	2.24	2.31	1.95	2.05
PFJM	1.26	1.65	2.14	2.31	2.09	1.85	1.98
TNC	1.58	2.38	1.93	2.13	2.20	1.83	1.98
TNTP	1.68	1.58	2.08	2.08	2.30	1.95	1.98
TNPFJM	1.45	1.60	1.98	2.23	2.13	1.80	1.95
HSC	1.40	1.68	2.28	2.40	2.58	1.98	2.18
HSTP	1.40	1.75	2.40	2.40	2.33	1.95	2.13
HSPFJM	1.08	1.70	2.30	2.40	2.05	1.90	2.00
SEM	0.07	0.05	0.04	0.03	0.02	0.02	0.02
*p*-value							
HS	0.0560	0.2010	0.0000	0.0010	0.0190	0.0410	0.0030
P	0.2330	0.0150	0.3480	0.6160	0.0000	0.1270	0.1160
HS*P	0.8330	0.0060	0.9890	0.6160	0.0010	0.2830	0.2940

HF: week; TN: averages under normal environmental conditions; HS: averages under temperature stress conditions; C: control group averages; TP: averages of commercial probiotics; PFJM: averages of probiotics obtained by adding 5% molasses to grass forage and incubating for 5 days; TNC: control group under normal environmental conditions; TNTP: commercial probiotics under normal environmental conditions; TNPFJM: probiotics obtained by adding 5% molasses to grass forage and incubating for 5 days under normal environmental conditions; HSC: control group under temperature environmental conditions; HSTP: commercial probiotics under temperature environmental conditions; HSPFJM: probiotics obtained by adding 5% molasses to grass forage and incubating for 5 days under temperature environmental conditions; *: interaction; P: effect of probiotics (TP and PFJM); HS*P: interaction between heat stress conditions and probiotics.

**Table 8:** Average pre-slaughter live weights, carcass weights, and internal organ weight percentages of the group.

	CA	Carcass (%)	Gizzard (%)	Liver (%)	Heart (%)	Spleen (%)	Bursa Fabricius (%)	Wing (%)	Back (%)	Thigh (%)	Breast (%)	Head (%)	Leg (%)	Abd. Fat (%)	Pancreas (%)
TN	2493.08	74.77	1.33	1.99	0.7	0.13	0.15	7.72	19.03	20.11	28.56	2.22	3.69	1.06	0.22
HS	2346.41	76.21	1.28	1.88	0.59	0.1	0.11	8.26	20.41	20.15	26.59	2.48	3.81	0.96	0.2
C	2121.9	76.2	1.44	1.99	0.69	0.12	0.14	8.29	19.71	20.43	27.05	2.43	3.81	0.92	0.23
TP	2507.57	74.72	1.24	1.93	0.64	0.12	0.14	7.81	19	20	29.33	2.19	3.76	1.12	0.2
PFJM	2661.19	75.25	1.25	1.92	0.63	0.11	0.13	7.76	20.14	19.95	26.76	2.38	3.67	1.01	0.2
TNC	2242.08	75.95	1.35	2.11	0.75	0.14	0.17	8	19	20.58	27.5	2.42	3.83	0.95	0.24
TNTP	2504.17	72.61	1.3	1.93	0.68	0.14	0.16	7.67	17.92	20.33	30.25	2.25	3.75	1.22	0.21
TNPFJM	2733	75.77	1.33	1.94	0.68	0.1	0.12	7.5	20.17	19.42	27.92	2	3.5	1	0.2
HSC	1961.67	76.54	1.56	1.84	0.62	0.11	0.09	8.67	20.67	20.22	26.44	2.44	3.78	0.88	0.22
HSTP	2512.11	77.53	1.15	1.92	0.58	0.09	0.11	8	20.44	19.56	28.11	2.11	3.78	0.99	0.19
HSPFJM	2565.44	74.56	1.14	1.89	0.56	0.11	0.14	8.11	20.11	20.67	25.22	2.89	3.89	1.01	0.2
SEM	31.44	0.43	0.03	0.04	0.02	0.01	0.01	0.13	0.31	0.3	0.44	0.05	0.08	0.04	0.01
*p*-value															
HS	0.023	0.097	0.523	0.202	0.001	0.045	0.01	0.035	0.028	0.951	0.028	0.014	0.459	0.245	0.38
P	0	0.46	0.009	0.828	0.23	0.546	0.942	0.159	0.43	0.807	0.038	0.068	0.851	0.17	0.106
HS*P	0.178	0.015	0.037	0.415	0.884	0.2	0.011	0.843	0.222	0.354	0.738	0	0.494	0.457	0.797

CA: live weight; %: percentage; TN: averages under normal environmental conditions; HS: averages under temperature stress conditions; C: averages of control group; TP: averages of commercial probiotics; PFJM: averages of probiotics obtained by adding 5% molasses to grass forage and incubating for 5 days; TNC: control group under normal environmental conditions; TNTP: commercial probiotics under normal environmental conditions; TNPFJM: probiotics obtained by adding 5% molasses to grass forage and incubating for 5 days under normal environmental conditions; HSC: control group under temperature environmental conditions; HSTP: commercial probiotics under temperature environmental conditions; HSPFJM: probiotics obtained by adding 5% molasses to grass forage and incubating for 5 days under temperature environmental conditions; Abd: abdominal fat; *: interaction; P: effect of probiotics (TP and PFJM); HS*P: interaction between heat stress conditions and probiotics.

**Table 9:** Average blood biochemical parameters of the groups.

	Glucose (mg/dL)	Total Protein (g/dL)	Albumin (mg/dL)	Uric Acid (mg/dL)	Triglycerides (mg/dL)	ALP (Unit/L)	Cholesterol (mg/dL)	HDLC (mg/dL)	LDLC (mg/dL)	VLDLC (mg/dL)
TN	231.19	2.87	1.45	6.44	42.13	2178.38	144.13	88.25	43.44	20.75
HS	223.85	3.06	1.52	6.65	39.90	1986.05	145.15	88.25	42.85	22.05
C	229.64	2.97	1.48	6.61	39.86	2010.93	146.50	87.64	43.21	21.00
TP	221.50	3.05	1.52	6.73	39.58	2275.42	142.42	86.33	43.08	21.83
PFJM	230.30	2.88	1.47	6.29	43.90	1911.70	144.90	91.40	43.00	21.70
TNC	231.40	2.62	1.32	6.06	42.40	2015.00	147.60	89.20	43.00	20.60
TNTP	226.00	2.90	1.47	6.44	39.60	2713.00	144.80	87.40	44.20	21.00
TNPFJM	235.33	3.05	1.54	6.77	44.00	1869.00	140.67	88.17	43.17	20.67
HSC	228.67	3.17	1.56	6.91	38.44	2008.67	145.89	86.78	43.33	21.22
HSTP	218.29	3.16	1.56	6.93	39.57	1962.86	140.71	85.57	42.29	22.43
HSPFJM	222.75	2.63	1.36	5.58	43.75	1975.75	151.25	96.25	42.75	23.25
SEM	1.06	0.05	0.02	0.05	0.62	30.06	1.44	1.49	0.72	0.38
*p*-value										
HS	0.001	0.173	0.244	0.615	0.26	0.001	0.584	0.672	0.648	0.052
P	0.008	0.232	0.234	0.001	0.025	0	0.479	0.312	0.987	0.497
HS*P	0.187	0.001	0.001	0	0.328	0	0.12	0.314	0.8	0.588

TN: averages under normal environmental conditions; HS: averages under temperature stress conditions; C: averages of control group; TP: averages of commercial probiotics; PFJM: averages of probiotics obtained by adding 5% molasses to grass forage and incubating for 5 days; TNC: control group under normal environmental conditions; TNTP: commercial probiotics under normal environmental conditions; TNPFJM: probiotics obtained by adding 5% molasses to grass forage and incubating for 5 days under normal environmental conditions; HSC: control group under temperature environmental conditions; HSTP: commercial probiotics under temperature environmental conditions; HSPFJM: probiotics obtained by adding 5% molasses to grass forage and incubating for 5 days under temperature environmental conditions; *: interaction; P: effect of probiotics (TP and PFJM); HS*P: interaction between heat stress conditions and probiotics; ALP: Alkaline Phosphatase (Unit/L); HDLC; high-density lipoprotein cholesterol; LDLC; low-density lipoprotein cholesterol; VLDLC; very-low-density lipoprotein cholesterol.

**Table 10:** pH, microbial populations, and organic acid levels of the gut microbiota in the groups.

	Duodenum pH	Cecum pH	Cecum Total Mesophilic Aerobic Bacteria Log_10_ cfu/mL (TAB)	Cecum Lactic Acid Bacteria Log_10_ cfu/mL (LAB)	Cecum *Enterobacter* Log_10_ cfu/mL	Cecum Coliform Log_10_ cfu/mL	Cecum *E. coli* Log_10_ cfu/mL	Cecum *E. coli* Log_10_ cfu/mL	Cecum Mold Log_10_ cfu/mL	Cecum Lactic Acid (LA)	Cecum Acetic Acid (AA)	Cecum Propionic Acid (PA)
TN	6.46	7.52	9.39	10.65	7.75	6.08	6.53	3.58	2.83	39.91	20.85	3.18
HS	6.52	7.52	9.22	9.79	8.07	5.78	4.84	3.21	2.74	24.51	15.20	3.63
C	6.48	7.60	9.31	9.77	8.00	5.76	6.73	4.26	2.84	33.27	16.35	2.57
TP	6.54	7.54	9.27	10.14	7.77	6.34	5.31	3.17	2.64	43.78	25.66	5.75
PFJM	6.45	7.42	9.34	10.75	7.95	5.67	5.02	2.76	2.88	19.57	12.06	1.89
TNC	6.47	7.55	9.10	10.45	7.82	6.15	6.83	4.11	2.83	40.18	16.84	2.41
TNTP	6.43	7.45	9.42	10.83	7.56	6.74	6.72	3.15	2.74	62.45	33.55	5.24
TNPFJM	6.48	7.55	9.63	10.69	7.87	5.34	6.04	3.48	2.91	17.11	12.16	1.88
HSC	6.49	7.65	9.52	9.09	8.19	5.38	6.64	4.40	2.84	26.37	15.86	2.72
HSTP	6.65	7.63	9.11	9.46	7.98	5.95	3.90	3.20	2.53	25.12	17.78	6.26
HSPFJM	6.42	7.28	9.04	10.81	8.04	6.00	3.99	2.04	2.85	22.03	11.97	1.90
SEM	0.01	0.01	0.01	0.00	0.00	0.01	0.00	0.01	0.00	0.02	0.02	0.04
*p*-value												
HS	0.018	0.89	0.000	0.000	0.000	0.000	0.000	0.000	0.000	0.000	0.000	0.000
P	0.017	0.000	0.008	0.000	0.000	0.000	0.000	0.000	0.000	0.000	0.000	0.000
HS*P	0.001	0.000	0.000	0.000	0.000	0.000	0.000	0.000	0.000	0000	0.000	0.002

TN: averages under normal environmental conditions; HS: averages under temperature stress conditions; C: averages of control group; TP: averages of commercial probiotics; PFJM: averages of probiotics obtained by adding 5% molasses to grass forage and incubating for 5 days; TNC: control group under normal environmental conditions; TNTP: commercial probiotics under normal environmental conditions; TNPFJM: probiotics obtained by adding 5% molasses to grass forage and incubating for 5 days under normal environmental conditions; HSC: control group under temperature environmental conditions; HSTP: commercial probiotics under temperature environmental conditions; HSPFJM: probiotics obtained by adding 5% molasses to grass forage and incubating for 5 days under temperature environmental conditions; *: interaction; P: effect of probiotics (TP and PFJM); HS*P: interaction between heat stress conditions and probiotics; TAB; total mesophilic aerobic bacteria; LAB; lactic acid bacteria.

**Table 11:** Correlation between organic acid concentrations and gut microbiota composition.

		Cecum TAB	Cecum LAB	Cecum Enterobacter	Cecum Coliform	Cecum *E. coli*	Cecum Yeast	Cecum Mold	Cecum Lactic Acid (LA)	Cecum Acetic Acid (AA)	Cecum Propionic Acid (PA)
Cecum TAB	pc	1	−0.079	−0.084	−0.434	0.580	0.446	0.414	−0.011	0.131	−0.164
	*p*		0.881	0.875	0.390	0.228	0.375	0.414	0.983	0.805	0.756
Cecum LAB	pc		1	−0.671	0.465	0.065	−0.551	0.414	0.304	0.183	−0.298
	*p*			0.145	0.353	0.902	0.257	0.415	0.558	0.729	0.566
Cecum Entero	pc			1	−0.753	−0.376	0.122	0.087	−0.786	−0.756	−0.336
	*p*				0.084	0.463	0.818	0.870	0.064	0.082	0.515
Cecum Coliform	pc				1	0.056	−0.342	−0.371	0.861 *	0.795	0.511
	*p*					0.916	0.507	0.469	0.027	0.059	0.301
Cecum *E. coli*	pc					1	0.733	0.479	0.517	0.387	−0.231
	*p*						0.097	0.337	0.294	0.448	0.660
Cecum Yeast	pc						1	0.125	0.091	0.028	−0.064
	*p*							0.813	0.864	0.958	0.905
Cecum Mold	pc							1	−0.182	−0.358	−0.930 **
	*p*								0.729	0.486	0.007
Cecum Lactic Acid (LA)	pc								1	0.938 **	0.445
	*p*									0.006	0.376
Cecum Acetic Acid (AA)	pc									1	0.649
	*p*										0.163
Cecum Propionic Acid (PA)	pc										1

PC: Pearson correlation; *: correlation is significant at 0.05 level; **: correlation is significant at 0.01 level; TAB, total mesophilic aerobic bacteria; LAB; lactic acid bacteria.

## Data Availability

The original contributions presented in this study are included in the article. Further inquiries can be directed to the corresponding author.
